# The health-related quality of life among survivors with post-COVID conditions in the United States

**DOI:** 10.1371/journal.pone.0320721

**Published:** 2025-05-05

**Authors:** Seyyed Sina Hejazian, Ajith Vemuri, Alireza Vafaei Sadr, Shima Shahjouei, Sasan Bahrami, Zhou Shouhao, Vida Abedi, Ramin Zand

**Affiliations:** 1 Department of Neurology, College of Medicine, The Pennsylvania State University, Hershey, Pennsylvania, United States of America; 2 Department of Public Health Sciences, College of Medicine, Pennsylvania State University, Hershey, Pennsylvania, United States of America; 3 Department of Neurology, Penn State Health Milton S. Hershey Medical Center, Hershey, Pennsylvania, United States of America; Kyung Hee University School of Medicine, KOREA, REPUBLIC OF

## Abstract

**Background:**

Even after a mild initial SARS-CoV-2 infection, a considerable proportion of patients experience long-lasting symptoms. However, there is scarce data on how post-COVID conditions (PCCs) are associated with health-related quality of life (HRQL) among COVID survivors. We aimed to study this association among adult COVID survivors in the United States.

**Method:**

The Behavioral Risk Factor Surveillance System 2022 data was utilized. The study population consisted of participants with a history of SARS-CoV-2 infection, categorized based on whether they had any PCCs. We evaluated the respondents’ HRQL in the two groups according to 1) self-reported general health (SRGH), 2) self-reported mental health, 3) self-reported physical health, and 4) efficiency in daily activities.

**Results:**

A total of 108,237 COVID survivors were included (35% were 18–34 years old and 46.5% were male), among whom 22.7% had PCCs. Unfavorable SRGH was more common among COVID survivors with PCCs than those without PCCs (25.7% vs. 15.5%, p < 0.001). Also, rates of having unfavorable mental and physical health and compromised daily efficiency for more than 13 days a month were significantly higher among PCC-positive respondents (p < 0.001). In the regression analysis adjusted for sociodemographics, comorbidities, and behavioral determinants of health, the presence of PCCs appeared as independent associates of unfavorable SRGH (aOR: 1.39, CI95%: [1.28–1.52], p < 0.001). Among PCC-positive respondents, dizziness on standing, mood changes, and musculoskeletal pain accompanied the highest odds of unfavorable HRQL. Based on a multivariate logistic regression analysis, early middle age, obesity, physical inactivity, diabetes, cardiovascular and pulmonary diseases, cancer, depression, smoking, being single and less educated, and having low annual income were independent factors associated with increased odds of unfavorable SRGH among survivors with PCCs.

**Conclusion:**

Our study corroborates that survivors with PCCs may experience significant adverse impacts on their health and daily life activities. Our results highlight the importance of further studies on PCCs’ diagnosis, follow-up, and treatment.

## 1. Introduction

COVID-19 continues to impose a significant burden on the survivors by post-COVID conditions (PCCs), even after a mild initial infection [1–5]. According to the most accepted definition, PCCs refer to any sign and symptom that appears or continues after the initial COVID-19 disease, remaining for at least 90 days [6]. The rate of PCCs varies from 10% to 80% across different countries and studies [7]. Our recent study estimated this number to be about 25% in the United States [1].

Investigating the impact of PCCs on health-related quality of life (HRQL) helps us better understand the burden of these conditions and plan effective rehabilitative strategies. While some studies have proposed adverse health impacts of PCCs [2,3,8,9], others have reported the opposite [10]. Besides these discrepancies, the majority of these studies are confined to a small study sample and risk of bias [7,11,12]. Therefore, our understanding of the HRQL-related impact of PCCs is still insufficient.

In this study, we aimed to evaluate the impact of long-term PCCs on the HRQL in the US using the BRFSS 2022, a comprehensive dataset representative of the US society [13]. We also studied potential factors associated with unfavorable HRQL among individuals with PCCs.

## 2. Methods

### 2.1. Design

The current study is based on the BRFSS 2022, a publicly available dataset from the Centers for Disease Control and Prevention (CDC) [13]. The BRFSS results are derived from an annual survey among US adults (aged 18 years or above) using both landline and mobile phone numbers [14]. The CDC uses complex sampling methods and survey weights in the BRFSS to deal with the non-response rate (55% in BRFSS 2022) and make it a true representative of the US society [15]. Multiple studies have shown the validity and reliability of BRFSS measures using weighted rates [16,17]. Calls are made during morning and evening hours every day of the week to avoid selection bias. Besides, misclassification errors are mitigated by directly inspecting the calls and a second call for randomly chosen phone numbers. The data in the BRFSS is self-reported by the respondents (except for the calculated variables, which are calculated based on the participants’ self-reported responses). All data in the BRFSS is anonymized. We complied with the Strengthening the Reporting of Observational Studies in Epidemiology guidelines for cross-sectional analyses [18].

### 2.2. Study population

Our study population consisted of COVID survivors who had ever experienced a health professional-confirmed SARS-CoV-2 infection. We excluded individuals with missing COVID-19, long-term PCCs, and self-reported general health data. We also excluded COVID-19 cases that were diagnosed only based on rapid home tests. A detailed study population flowchart is presented in S1 Fig in S1 File. We performed a complete case analysis based on 108,237 COVID survivors, representing a weighted number [19] of 70,002,617 adults in the US.

### 2.3. Evaluating long-term post-COVID conditions

Consistent with the definition provided by the Department of Health and Human Services (HHS) in collaboration with CDC and other partners [6], the BRFSS defines long-term PCCs as “symptoms lasting three months or longer that did not exist prior to having coronavirus infection.” These conditions may or may not be directly associated with the SARS-COV2 viral infection [20]. Besides, all COVID survivors with long-term PCCs were questioned about their primary post-COVID symptoms. Table 1 summarizes the questions and potential answers utilized in the BRFSS to gather the relevant data.

**Table 1:** HRQL-related Questions in the BRFSS and Their Possible Answers.

Variable		Question	Possible answers
**Long-term Post-COVID Conditions**	Positive post-COVID conditions	Did you have any symptoms lasting 3 months or longer that you did not have prior to having coronavirus or COVID-19?	(1) No (2) Yes
	Main post-COVID symptom	Which of the following was the primary symptom that you experienced? Was it ….	(1) Fatigue (2) brain fog^*^ (3) dyspnea (4) musculoskeletal pain (5) heart palpitation (6) dizziness on standing (7) mood disorders (8) loss of taste or smell (9) post-physical (10) post-exertional malaise ^**^ (11) other symptoms (12) No activity-limiting symptom
**Health-related Quality of Life**	Self-reported General Health	Would you say that, in general, your health is:	(1) Excellent (2) Very good (3) Good (4) Fair (5) Poor
	Mental Health	Thinking about your mental health, which includes stress, depression, and problems with emotions, for how many days during the past 30 days was your mental health not good?	0 to 30 days
	Physical Health	Thinking about your physical health, which includes physical illness and injury, for how many days during the past 30 days was your physical health not good?	0 to 30 days
	Daily Efficiency	During the past 30 days, for about how many days did poor physical or mental health keep you from doing your usual activities, such as self-care, work, or recreation?	0 to 30 days

*Brain fog refers to difficulty thinking, concentrating, or forgetfulness.

**Post-exertional malaise is the other term for the worsening of the symptoms after physical or mental activity [15].

### 2.4. Assessment of health-related quality of life

This study’s outcome of interest was evaluating the impact of PCCs on health-related quality of life (HRQL). The BRFSS contains four variables that assess the respondents’ HRQL. These variables include self-reported general health (SRGH), mental health, physical health, and daily efficiency. We further expanded the impact of PCCs on SRGH as a subcomponent of HRQL in our analysis. Table 1 lists the exact questions and potential replies for each HRQL-related variable. In the current paper, “excellent,” “very good,” and “good” SRGH are categorized as “Favorable,” while “fair” and “poor” SRGH are considered “Unfavorable.” Following the BRFSS, we classified the other three HRQL-related variables (mental health, physical health, and daily efficiency) as zero days in a month, one to 13 days in a month, and more than 13 days in a month.

### 2.5. Other studied characteristics

We also examined the target population’s sociodemographics, comorbidities, and behavioral determinants of health. Following is the list of sociodemographics: age, sex, marital status, race and ethnicity, education level, annual household income (AHHI), health insurance coverage, state, and rurality. Age is classified as early adulthood (18–34 years old), early middle age (35–44 years old), late middle age (45–64 years old), and late adulthood (>64 years old) [21]. Health insurance includes both public and private insurance. Rural areas are defined based on the US Office of Management and Budget standards [22].

Comorbidities included diabetes, heart disease, pulmonary disease, depression, kidney disease, arthritis, cancer, and obesity. Individuals with a body mass index (BMI) of 30 Kg/m2 or higher are considered obese. Behavioral determinants of health consisted of cigarette or e-cigarette smoking, heavy alcohol users, exercise or physical activity, and sleep hygiene. To classify individuals as smokers, the standard criterion employed was a lifetime total of 100 cigarettes smoked. However, the only people who qualified as current smokers were those who had smoked at least one cigarette in the 30 days before the survey phone call. Only activities other than daily jobs were considered to determine if an individual was physically active. Habitual sleep for less than 7 hours or more than 9 hours were thresholds for short and long sleep, respectively [23]. The information about the respondents’ smoking status, physical activity, and daily sleep pertains only to the one month before the survey phone call. We followed the BRFSS recommendations to categorize all study variables [15]. The details of each study variable are summarized in S1 and S2 Tables in S1 File.

### 2.6. Statistical analysis

Data imputation was carried out for two variables with missing rates above 5%, including AHHI and BMI (INCOMG1 and BMI5CAT variables in the BRFSS dataset, respectively), using a sub-dataset of COVID survivors. To address the missing data, iterative imputation random forest classification and regression models were employed for categorical and continuous variables, respectively [24,25]. For this purpose, the INCOMG1 classes were encoded as: ‘1: $<15k’, ‘2: $15–25k’, ‘3: $25–35k’, ‘4: $35–50k’, ‘5: $50-100k’, ‘6: $100-200k’, ‘7:>200k’. Similarly, BMI5CAT categories were encoded as ‘1: <18.5 kg/m^2^’, ‘2: 18.5-25 kg/m^2^’, ‘3: 25-30 kg/m^2^’, and ‘4: >30 kg/m^2^’. We carried out a holdout analysis [26], randomly withholding 50 existing values and iterating the imputation process ten times to evaluate the imputation performance. The observed accuracy results were 0.986 (CI95%: [0.964, 0.997]) for INCOMG1 and 0.996 (CI95%: [0.980, 0.999]) for BMI5CAT. The imputation was performed in Python 3.11.5 using scikit-learn library version 1.3.2.

Statistical analyses were performed using R 4.3.2 (R Core Team, Vienna, Austria). We utilized survey-specific R functions (from survey and srvyr packages) to include survey design, survey strata, and sampling weights in our analysis. The Rao-Scott χ2 test was used to calculate the significance of the difference between study groups. Multivariate logistic regression models evaluated the association between PCCs and the SRGH among COVID survivors. Logistic regression was also used to determine the factors associated with unfavorable SRGH among COVID survivors with PCCs. These models were incrementally adjusted for socio-demographics, comorbidities, and behavioral determinants of health. Two other versions of the dataset were used to perform sensitivity analyses of the logistic regression models. The first was a dataset with non-imputed variables, and the second was a dataset with missing data as a separate category. Figures were plotted with GraphPad Prism 9.4.1.681. State-wise prevalence of long-term PCC was visualized using Datawrapper, accessed on April 16, 2024 (https://www.datawrapper.de/maps/choropleth-map). P-values lower than 0.05 are significant.

## 3. Results

### 3.1. General characteristics of COVID survivors

Overall, 108237 COVID survivors were studied (46.5% male), most of whom were in their early adulthood (35%) or late middle age (31%). The prevalence of obesity was 37.3%. Over half of COVID survivors were non-Hispanic White (58.2%), had a university degree (63.4%), and earned more than 50k a year (60.9%). Among comorbidities, arthritis (25.5%), depression (23.7%), and pulmonary disease (21.2%) were the most common ones. Current or former cigarette smoking was slightly more prevalent than current or former e-cigarette smoking (33.8% vs. 31.6%). Physical activity and adequate sleep were evident among 77.5% and 58.1% of the study population, respectively (Table 2 and S2 Table in S1 File).

**Table 2:** General Characteristics of Study Population by Having Post-COVID Condition.

Feature	COVID Survivors (CI95% of percentage)	COVID Survivors with PCCs (CI95% of percentage)	COVID Survivors without PCCs (CI95% of percentage)	P-value
Respondents, raw unweighted frequency	108237	25216	83021	–
Respondents, raw US weighted frequency	70002617	15877641	54124977	–
**Age**				
Early adulthood (18–34 years old)	35% (34.4-35.5)	32% (30.9-33.2)	35.8% (35.1-36.5)	**<0.001**
Early middle age (35–44 years old)	18.9% (18.4-19.3)	20.4% (19.4-21.5)	18.4% (17.9-18.9)	
Late middle age (45–64 years old)	31% (30.4-31.5)	33.8% (32.7-35)	30.1% (29.5-30.8)	
Late adulthood (>64 years old)	15.2% (14.8-15.6)	13.7% (12.9-14.5)	15.6% (15.2-16.1)	
**Sex**				
Male	46.5% (45.9-47.1)	36.8% (35.6-38)	49.4% (48.7-50.1)	**<0.001**
Female	53.5% (52.9-54.1)	63.2% (62-64.4)	50.6% (49.9-51.3)	
**Body mass index (BMI)**				
<18.5 Kg/m2 (Underweight)	1.6% (1.4-1.7)	1.6% (1.2-2)	1.5% (1.4-1.7)	**<0.001**
18.5-24.9 Kg/m2 (Normal weight)	26.8% (26.3-27.4)	22.6% (21.6-23.6)	28.1% (27.4-28.7)	
25-29.9 Kg/m2 (Overweight)	34.3% (33.7-34.9)	31.5% (30.4-32.7)	35.1% (34.5-35.8)	
≥30 Kg/m2 (Obese)	37.3% (36.7-37.9)	44.3% (43-45.5)	35.3% (34.6-35.9)	
**Marital status** ^#^				
Single	48.7% (48.1-49.3)	49.9% (48.6-51.1)	48.3% (47.6-49)	**0.035**
Married	51.3% (50.7-51.9)	50.1% (48.9-51.4)	51.7% (51-52.4)	
**Race and ethnicity**				
Hispanic	20.3% (19.8-20.9)	20.5% (19.3-21.7)	20.3% (19.7-20.9)	**<0.001**
Non-Hispanic, White	58.2% (57.6-58.8)	60.3% (59-61.6)	57.6% (56.9-58.3)	
Non-Hispanic Black	11.2% (10.8-11.6)	10.2% (9.4-11)	11.5% (11.1-12)	
Non-Hispanic, Asian	5.3% (4.9-5.7)	2.8% (2.2-3.3)	6% (5.6-6.5)	
Non-Hispanic, American Indian, or Alaskan Native	1.2% (1.1-1.3)	1.6% (1.3-1.8)	1.1% (1-1.2)	
Non-Hispanic, Native Hawaiian, or other Pacific Islanders	0.4% (0.3-0.5)	0.3% (0.2-0.5)	0.4% (0.4-0.5)	
non-Hispanic, Multiracial	3.3% (3.1-3.6)	4.3% (3.7-4.9)	3% (2.8-3.3)	
**Education**				
less than high school degree	9.5% (9.1-10)	10.2% (9.2-11.3)	9.3% (8.8-9.8)	0.121
High school degree	27.1% (26.5-27.6)	26.4% (25.3-27.5)	27.3% (26.7-27.9)	
Higher than high school degree	63.4% (62.8-64)	63.4% (62.1-64.6)	63.4% (62.7-64.1)	
**Annual household income (AHHI)**				
$ < 25k	12.5% (12.1-12.9)	15.2% (14.3-16.2)	11.7% (11.2-12.2)	**<0.001**
$25k-50k	26.7% (26.1-27.2)	27.9% (26.8-29)	26.3% (25.7-26.9)	
$50k-100k	30.6% (30-31.1)	31.5% (30.4-32.7)	30.3% (29.7-30.9)	
$ > 100k	30.3% (29.7-30.8)	25.4% (24.3-26.4)	31.7% (31.1-32.3)	
**Rural residence**				
Urban areas	93.9% (93.7-94.1)	93.3% (92.8-93.7)	94.1% (93.9-94.3)	**0.001**
Rural areas	6.1% (5.9-6.3)	6.7% (6.3-7.2)	5.9% (5.7-6.1)	
**Health Insurance Coverage**				
With health insurance	93% (92.6-93.3)	91.9% (91.2-92.7)	93.3% (92.9-93.7)	**0.002**
Without health insurance	7% (6.7-7.4)	8.1% (7.3-8.8)	6.7% (6.3-7.1)	
**Comorbidities**				
Diabetes ^ф^	11.5% (11.2-11.9)	13.3% (12.5-14.1)	11% (10.6-11.5)	**<0.001**
Heart disease ^£^	5.8% (5.6-6.1)	7.4% (6.9-7.9)	5.3% (5.1-5.6)	**<0.001**
Pulmonary disease ^⁋^	21.2% (20.7-21.7)	29.7% (28.6-30.8)	18.8% (18.2-19.3)	**<0.001**
Depression	23.7% (23.2-24.2)	34% (32.8-35.2)	20.7% (20.2-21.2)	**<0.001**
Kidney disease	3.6% (3.4-3.8)	4.7% (4.3-5.2)	3.2% (3-3.5)	**<0.001**
Arthritis	25.5% (25-26)	33.4% (32.3-34.5)	23.2% (22.6-23.7)	**<0.001**
Cancer	7.2% (6.9-7.5)	7.8% (7.2-8.4)	7% (6.6-7.3)	**0.014**
Stroke	3.2% (2.9-3.4)	4.2% (3.8-4.6)	2.8% (2.6-3.1)	**<0.001**
**Cigarette Smoking Status**				
Non-smoker	66.2% (65.7-66.8)	61.3% (60.1-62.5)	67.7% (67.1-68.3)	**<0.001**
Former smoker	23.6% (23.1-24.1)	26.5% (25.5-27.6)	22.7% (22.1-23.3)	
Current smoker	10.2% (9.8-10.6)	12.2% (11.4-13)	9.6% (9.2-10)	
Heavy alcohol drinker	6.9% (6.6-7.1)	7% (6.4-7.6)	6.8% (6.5-7.1)	0.529
**E-cigarette Smoking Status**				
Non-smoker	68.5% (67.9-69)	66.9% (65.7-68.1)	68.9% (68.3-69.6)	**0.005**
Former smoker	22.4% (21.9-22.9)	23.1% (22.1-24.2)	22.1% (21.6-22.7)	
Current smoker	9.2% (8.8-9.5)	10% (9.2-10.7)	8.9% (8.5-9.3)	
**Daily exercise or physical activity** ¥				
With physical activity	77.5% (77-78)	74.5% (73.4-75.6)	78.4% (77.9-79)	**<0.001**
Without physical activity	22.5% (22-23)	25.5% (24.4-26.6)	21.6% (21-22.1)	
**Daily Sleep Duration**				
7-9 hours/day (adequate sleepers)	58.1% (57.5-58.7)	50.9% (49.6-52.2)	60.2% (59.5-60.9)	**<0.001**
<7 hours/day (short sleepers)	38.9% (38.3-39.5)	45.7% (44.5-47)	37% (36.3-37.6)	
>9 hours/day (long sleepers)	3% (2.7-3.2)	3.4% (2.9-3.8)	2.9% (2.6-3.1)	

# Never-married, divorced, widowed, and unmarried couples are considered single. ^ф^ Diabetes does not include pre-diabetes or gestational diabetes.

£ Heart disease refers to myocardial infarction, angina, or coronary heart disease.

⁋ Pulmonary diseases refer to asthma, chronic obstructive pulmonary disease, emphysema, and chronic bronchitis.

- Percentages for any given characteristic might not add up to 100% due to the weighted nature of the estimates.

- Significant p-values (<0.05) are bolded.

The overall prevalence of PCCs was 22.7%. Comparing COVID survivors by having PCCs shows that those with PCCs were more likely to be in their middle ages, female, obese, Non-Hispanic White (p < 0.001), single (p = 0.035), and former or current smokers. Moreover, having lower income, lower physical activity (p < 0.001), and poor sleep hygiene, residing in rural areas (p = 0.001), and lacking medical insurance (p = 0.002) were more prevalent among those with PCCs. Comorbidities, including diabetes, cardiovascular, pulmonary, and kidney diseases, depression, arthritis (p < 0.001), and cancer (p = 0.014), were more likely to be reported among survivors with PCCs (Table 2).

### 3.2. The HRQL among COVID survivors with PCCs

**Fig 1:**
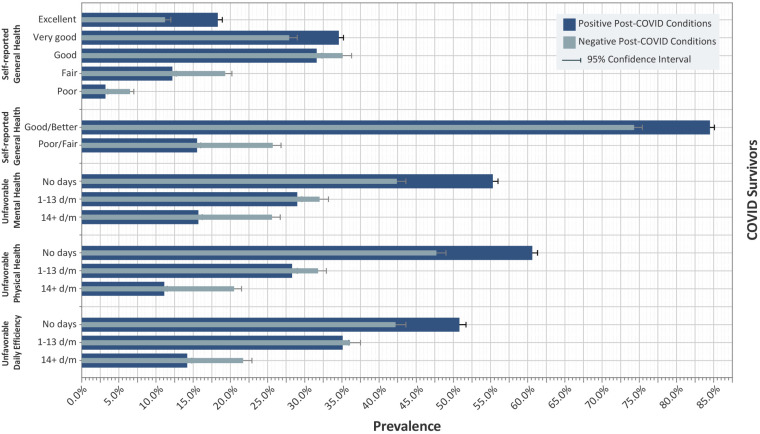
Self-reported general health and number of days in a month with “Not Good” mental health, physical health, and compromised daily efficiency among survivors with and without post-COVID conditions. d/m: days per month.

As mentioned earlier, the BRFSS assesses the respondents’ HRQL based on SRGH, mental and physical health, and daily efficiency. As Fig 1 and S3 Table in S1 File demonstrate, unfavorable SRGH was significantly more common among COVID survivors with PCCs compared to those without PCCs (25.7% vs. 15.5%, p < 0.001). Moreover, reporting no days with “Not Good” mental or physical health was more common among those without PCCs (p < 0.001). Besides, survivors with PCCs were more likely to report compromised daily efficiency due to “Not good” mental or physical health (p < 0.001).

### 3.3. Association between PCCs and unfavorable SRGH among COVID survivors

**Fig 2:**
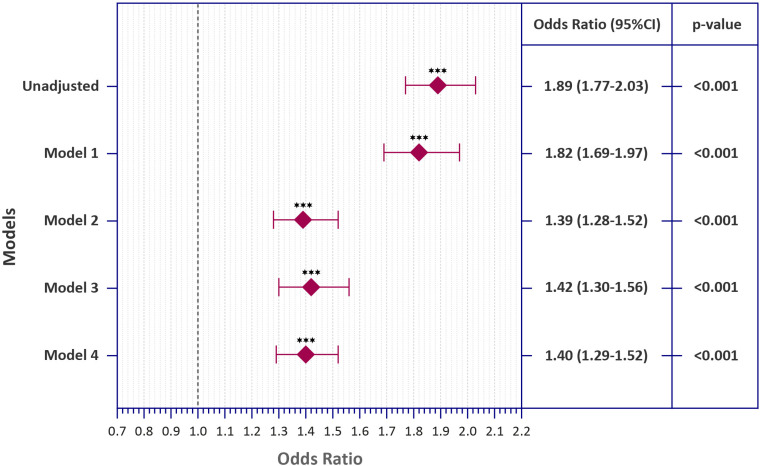
Logistic Regression Models for Evaluating the Association Between Post-COVID Conditions and “Unfavorable” Self-reported General Health Among Adult Survivors in the United States. “Model 1” was adjusted only for sociodemographics (Age, Sex, Marital Status, Race and Ethnicity, Education, AHHI, Rural residence, and Health insurance coverage). “Model 2” was adjusted for sociodemographics (Age, Sex, Marital Status, Race and Ethnicity, Education, AHHI, Rural residence, and Health insurance coverage), comorbidities (Diabetes, Cardiovascular diseases, Pulmonary diseases, Depression, Kidney disorders, Cancer, Arthritis, Obesity), and behavioral determinants of health (Cigarette smoking, E-cigarette smoking, Heavy alcohol consumption, Exercising, and Habitual sleep duration). “Model 3” was adjusted for all sociodemographics, comorbidities, and behavioral determinants of health using the non-imputed AHHI and BMI data. “Model 4” was adjusted for all sociodemographics, comorbidities, and behavioral determinants of health using the missing data as a separate category. *** indicates p < 0.001.

Multivariate logistic regression analysis showed that PCCs are associated with higher rates of unfavorable SRGH (cOR: 1.89, CI95%: [1.77–2.03], p < 0.001). The odds ratios were still significant after adjusting for only demographics (Model 1, aOR: 1.82, CI95%: [1.69–1.97], p < 0.001) or demographics, comorbidities, and behavioral determinants of health (Model 2, aOR: 1.39, CI95%: [1.28–1.52], p < 0.001). In sensitivity analyses, model 3 was defined using non-imputed data (aOR: 1.42, CI95%: [1.30–1.56], p < 0.001), and model 4 was created using a dataset with missing data classified as a separate category (aOR: 1.40, CI95%: [1.29–1.52], p < 0.001), which did not cause significant alterations in the results (Fig 2).

### 3.4. Prevalence of unfavorable SRGH among survivors with different post-COVID symptoms

**Fig 3:**
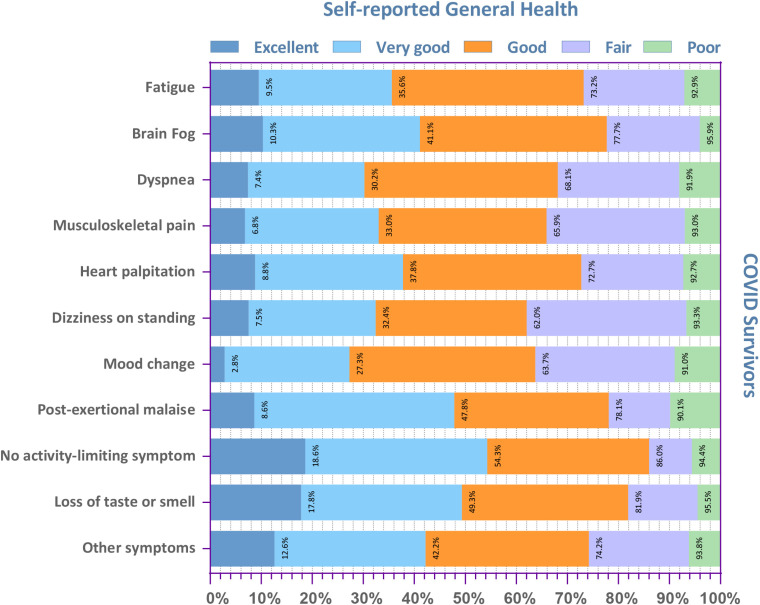
Self-reported General Health Among Survivors with Different Primary Post-COVID Symptoms.

Rates of unfavorable SRGH varied considerably across COVID survivors with different primary post-COVID symptoms. As pictured in Fig 3 and S4 Table in S1 File, dizziness on standing (38%), mood disorders (36.3%), and musculoskeletal pain (34.1%) were accompanied by the highest prevalence of unfavorable SRGH compared to other post-COVID symptoms. On the other hand, unfavorable SRGH was least common among those who had loss of taste or smell (18.1%), post-exertional malaise (21.9%), or brain fog (22.3%).

### 3.5. SRGH of COVID survivors by their age and sex

**Fig 4:**
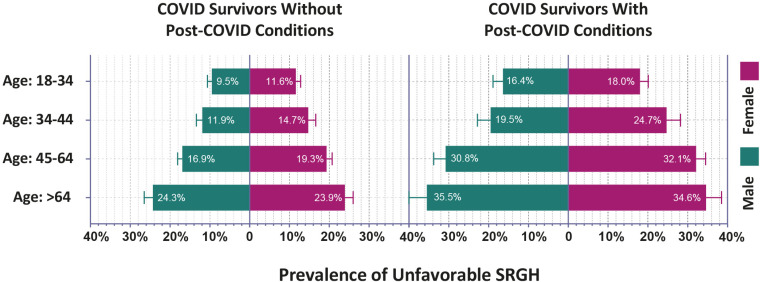
Age and Sex-wise Prevalence of Unfavorable Self-reported General Health Among COVID Survivors.

Age- and sex-wise discrepancies in the prevalence of unfavorable SRGH are shown in Fig 4 and S5 Table in S1 File. Among both men and women, older individuals had worse SRGH. In all age categories, the prevalence of unfavorable SRGH was more common among both sexes with PCCs than without PCCs. Only the differences between men and women COVID survivors without PCCs in the early middle age group (11.9% vs. 14.7%, p = 0.036) and men and women COVID survivors with PCCs in the early adulthood age group (16.4% vs. 18%, p < 0.001) were statistically significant.

### 3.6. State-wise prevalence of unfavorable SRGH among COVID survivors

**Fig 5:**
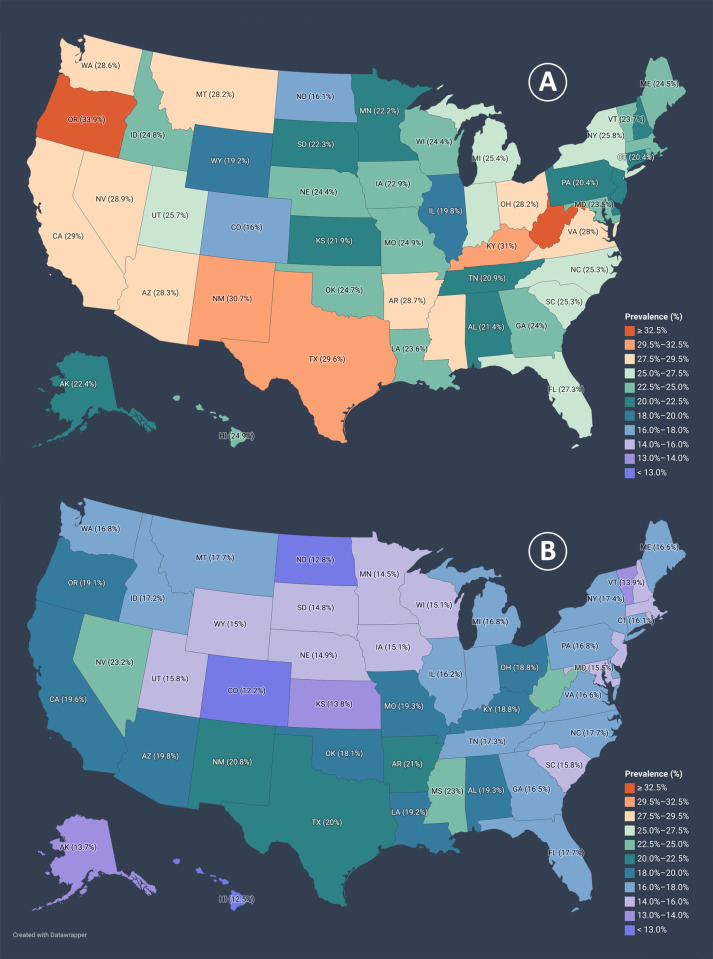
State-wise Prevalence of Unfavorable Self-reported General Health among COVID Survivors. (A) Shows the state-wise prevalence of unfavorable SRGH among COVID survivors with PCCs. (B) Shows the state-wise prevalence of SRGH among all COVID survivors. AL: Alabama, AK:Alaska, AZ: Arizona, AR: Arkansas, CA: California, CO: Colorado, CT: Connecticut, DE: Delaware, DC: District of Columbia, FL: Florida, GA: Georgia, HI: Hawaii, ID: Idaho, IL: Illinois, IN: Indiana, IA: Iowa, KS: Kansas, KY: Kentucky, LA: Louisiana, ME: Maine, MD: Maryland, MA: Massachusetts, MI: Michigan, MN: Minnesota, MS: Mississippi, MO: Missouri, MT: Montana, NE: Nebraska, NV: Nevada, NH: New Hampshire, NJ: New Jersey, NM: New Mexico, NY: New York, NC: North Carolina, ND: North Dakota, OH: Ohio, OK: Oklahoma, OR: Oregon, PA: Pennsylvania, RI: Rhode Island, SC: South Carolina, SD: South Dakota, TN: Tennessee, TX: Texas. UT: Utah. VT: Vermont, VA: Virginia, WA: Washington, WV: West Virginia, WI: Wisconsin, WY: Wyoming.

As illustrated in Fig 5 and S6 Table in S1 File, in all states, unfavorable SRGH was more common among individuals with PCCs. West Virginia had the highest prevalence of unfavorable SRGH among all COVID survivors and COVID survivors with PCCs (23.9% and 34.5%, respectively). Among all COVID survivors, Nevada (23.2%), Mississippi (23%), and Puerto Rico (22.6%) were the other three regions with high prevalences of unfavorable SRGH. On the other hand, among those with PCCs, Oregon (33.9%), Kentucky (31%), and New Mexico (30.7%) had the highest rates of unfavorable SRGH after West Virginia. Favorable SRGH was most common among COVID survivors of the District of Columbia (90.5%) and PCC-positive COVID survivors of the Virgin Islands (90.3%).

### 3.7. Factors associated with unfavorable SRGH among COVID survivors with PCCs

**Fig 6:**
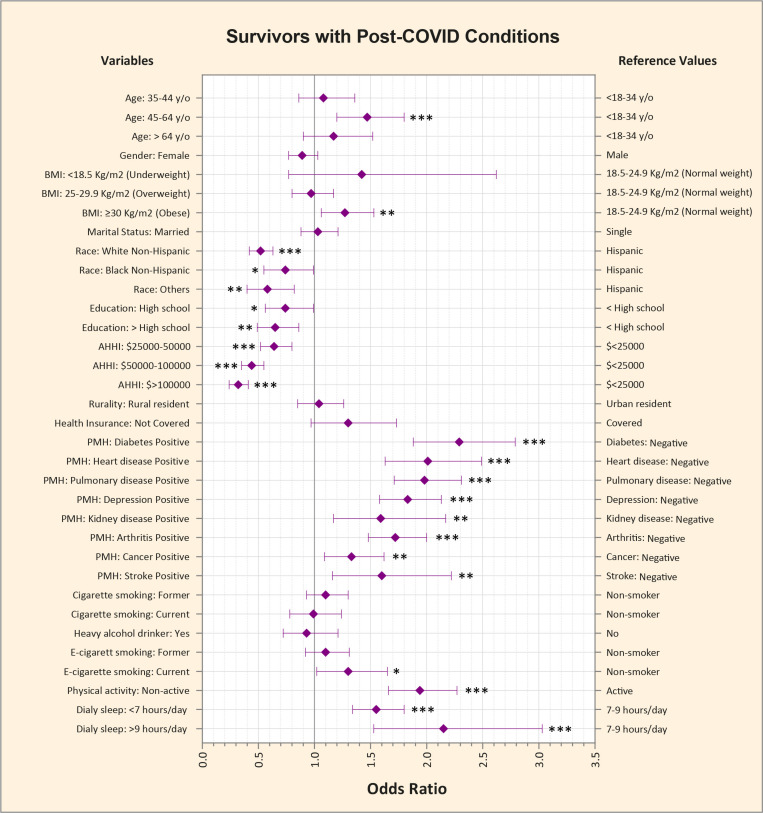
Multivariate Logistic Regression Analysis Showing Independent Factors Associated with Unfavorable Self-reported General Health among COVID Survivors with Post-COVID Conditions. The odds ratios were adjusted for sociodemographics (Age, Sex, Marital Status, Race and Ethnicity, Education, AHHI, Rural residence, and Health insurance coverage), comorbidities (Diabetes, Cardiovascular diseases, Pulmonary diseases, Depression, Kidney disorders, Cancer, Arthritis, Obesity), and behavioral determinants of health (Cigarette smoking, E-cigarette smoking, Heavy alcohol consumption, Exercising, and Habitual sleep duration). * Indicates p < 0.05, ** Indicates p < 0.01, *** Indicates p < 0.001.

S7 Table in S1 File compares the general characteristics of survivors with PCCs based on their SRGH. The independent factors associated with unfavorable SRGH among those with PCCs were investigated using multivariate logistic regression models adjusted for sociodemographics, comorbidities, and behavioral determinants of health (Fig 6 and S8 Table in S1 File). Compared to PCC-positive COVID survivors in their early adulthood, those in their late middle age were more likely to have unfavorable SRGH (aOR = 1.47, CI95%: [1.2–1.8], p < 0.001). Obese individuals had higher odds of having unfavorable SRGH compared to those with a BMI within the normal range (aOR = 1.27, CI95%: [1.06–1.53], p < 0.001). Unfavorable SRGH was more probable among Hispanics, less-educated individuals, and those with lower levels of AHHI (p < 0.001). Among comorbidities, Diabetes (aOR = 2.29, CI95%: [1.88–2.79], p < 0.001), heart disease (aOR = 2.01, CI95%: [1.63–2.49], p < 0.001), and pulmonary disease (aOR = 1.98, CI95%: [1.63–2.31], p < 0.001) had the most significant association with unfavorable SRGH. Moreover, active cigarette smokers (aOR = 1.3, CI95%: [1.02–1.65], p < 0.001), less active individuals (aOR = 1.94, CI95%: [1.66–2.27], p < 0.001), short sleepers (aOR = 1.55, CI95%: [1.34–1.8], p < 0.001), and long sleepers (aOR = 2.15, CI95%: [1.53–3.03], p < 0.001) had greater likelihood of having unfavorable SRGH.

## 4. Discussion

In this cross-sectional study in the US, we used a comprehensive dataset to evaluate long-term PCCs’ association with the HRQL, assessed by SRGH, mental and physical health, and daily efficiency (Table 1). We showed that HRQL is significantly worse among COVID survivors with PCCs than those without PCCs. These differences were consistent in mental and physical health as well as SRGH. We also demonstrated that the higher likelihood of unfavorable SRGH among COVID survivors with PCCs is consistent among both sex groups, in all age categories, and across all states. However, not all post-COVID symptoms had the same association with the SRGH of COVID survivors. Individuals with dizziness on standing, mood changes, and musculoskeletal pain had the highest rates of unfavorable SRGH, while loss of taste or smell, post-exertional malaise, and brain fog were less associated with unfavorable SRGH.

HRQL is an important factor, enabling us to assess the impact of diseases on patients’ daily lives [27]. The empirical findings in this study help us understand how the HRQL is impacted among those with PCCs, which is vital for providing an efficient care plan and rehabilitation strategy. Several prior smaller studies have highlighted the adverse effects of PCCs on HRQL and showed similar results [28–33]. Similar results patterns have been reported from various countries, including Japan [29], Turkey [34,35], and Korea [36]. The adverse impact of PCCs on the HRQL has also been reported using different health-related questionnaires, including EQ-5D [29,37,38] and SF-36 [39]. One study did not demonstrate any statistically significant differences in general HRQL between those with and without PCCs based on the SF-12 questionnaire [10]. Another study using self-reported data on the HRQL of international COVID survivors demonstrated that the PCCs-derived compromised HRQL could restrict the individuals’ capacity to perform daily activities [40].

Our analysis also corroborated that survivors with PCCs experience more days with poor mental or physical health compared to those without PCCs. Most studies evaluating the mental and physical health of COVID survivors have shown that PCCs impact physical health more severely than mental health [32,41,42]. Moreover, our finding of PCCs’ adverse effects on the daily efficiency of COVID survivors aligns with previous studies [7,43–48]. Some studies have shown that more severe and prolonged PCCs are associated with worse capacity for daily activities [49,50]. It has also been reported that individuals with PCCs have an increased likelihood of being unemployed or decreased odds of working full-time [47,48,51]. One study highlighted that the adverse negative impact of PCCs on the workability of women is more substantial than that of men [52]. It has been estimated that PCCs cause approximately $50 billion in annual salary loss in the UK [53].

Although the unfavorable HRQL was more common among women in this study, the multivariate regression model showed that sex is not an independent determinant of general HRQL among those with PCCs. This finding is consistent with many previous studies [42,54]. In contrast, some studies have reported opposite results [34,55,56]. According to a recent systemic review, female sex and aging are risk factors for impaired HRQL after SARS-CoV-2 infection [56]. Another study showed that compared to PCC-positive women, younger male COVID survivors are more prone to impaired mental health [34]. On the other hand, some studies have reported that despite no differences in mental or general health, women experience worse physical health due to PCCs [40–42]. We also showed that COVID survivors aged 45–64 years old are more susceptible to impaired general HRQL than those in their early adulthood. This finding contrasts some studies that have not reported age as a risk factor for impaired HRQL among PCC-positive individuals [40,41]. Such discrepancies in the association of sex and age with HRQL among those with PCCs highlight the need for future studies.

Variations in the association between the SRGH and different post-COVID symptoms show the importance of prioritizing healthcare services. Our previous analysis of the BRFSS 2022 showed that fatigue, dyspnea, and loss of taste or smell are the most common PCCs among COVID survivors [1]. However, the current study favors a more significant association between impaired SRGH and certain types of PCCs, including dizziness on standing, mood changes, and musculoskeletal pain. On the other hand, loss of taste or smell had the most negligible associations. These findings contrast with many other studies that have introduced fatigue as the most significant post-COVID symptom associated with compromised HRQL [3,34,57–60]. However, Soh et al. demonstrated that those with neuropsychiatric symptoms had the worst HRQL [36]. In another study by Kabir et al., musculoskeletal pain had the highest adverse effects on HRQL [61]. Based on previous studies, the number [40], duration [61], and severity [62] of post-COVID symptoms are negatively associated factors with the HRQL of COVID survivors, which may be the rationale for the contradictory results from different studies.

We also showed that the independent factors associated with increased odds of unfavorable SRGH among PCC-positive individuals include early middle ages, obesity, physical inactivity, comorbidities, cigarette smoking, daily sleep hygiene, being single, Hispanic, and less educated, and having low AHHI. According to our previous study, among these factors, obesity, low AHHI, comorbidities, cigarette smoking, short sleep, and being Hispanic are also associated with a higher prevalence of PCCs among COVID survivors [1]. In contrast, the association of age with the odds of having PCCs and impaired HRQL was contradictory. While having PCCs was more common among younger COVID survivors [1], the association of PCCs with impaired HRQL was more prominent among those in their late middle ages. Overall, these findings show that specific subpopulations of COVID survivors who experience PCCs may be more susceptible to impaired HRQL following COVID infection due to PCCs. Therefore, healthcare providers and policymakers can prioritize more individualized rehabilitation services following SARS-CoV-2 infection for at risk COVID survivors.

Similar to our findings, several studies have reported psychological impairments [57], low educational levels [3], higher BMI [62], comorbidities [56,62,63], low physical activity [41], and smoking [62] as risk factors for unfavorable HRQL among those with PCCs. In another study, psychosocial impairments were only associated with mental aspects of HRQL [34]. In contrast, Tak et al. found no association between BMI, health insurance, education, and HRQL [40]. They also showed that COVID survivors with lower physical activity before acute infection had better HRQL due to PCCs [40].

This study’s main strength is using a comprehensive dataset representative of the US population. However, our study had some limitations. 1) The BRFSS uses self-reported data and does not employ a structured questionnaire to assess participants’ HRQL. 2) Institutionalized individuals are not included in the BRFSS. 3) The time and severity of the first SARS-CoV-2 infection, medications used in this period, and the length of PCCs were not disclosed in the BRFSS. However, several studies have shown that PCCs-related impacts on the HRQL are regardless of the acute infections’ timing, severity, duration, or care setting [37,40,41,57,61,63]. 4) We had limited access to the COVID vaccination status of the participants. 5) The number of post-COVID symptoms included in the BRFSS dataset is limited. 6) Recall bias is a potential concern due to the time gap between experiencing PCC and completing the survey. Despite these limitations, the findings from our study make valuable contributions to the current literature and may potentially improve the follow-up and rehabilitation process of COVID survivors living with PCCs.

## 5. Conclusion

Our study confirms that PCCs are associated with higher rates of impaired HRQL and daily life activities. Additionally, factors such as early middle age, cigarette smoking, poor sleep hygiene, physical inactivity, lower educational attainment, obesity, low AHH, and comorbidities were associated with higher reports of unfavorable SRGH among individuals with PCCs. These findings emphasize the need for future research on chronic follow-up, cross-specialty support, and multidisciplinary rehabilitation for COVID survivors, particularly those with PCCs.

## Supporting information

S1 File Online Supplementary File Containing S1 Fig and S1-8 Tables. (PDF)

S2 File Whole dataset used for analysis in this study. (ZIP)
