# Supplementary material for: The health-related quality of life among survivors with post-COVID conditions in the United States
Source: PLoS One. 2025 May 5;20(5):e0320721. doi: 10.1371/journal.pone.0320721 (PMC12052100; doi:10.1371/journal.pone.0320721)
Supplement: S1 File — (PDF) [file pone.0320721.s001.pdf]

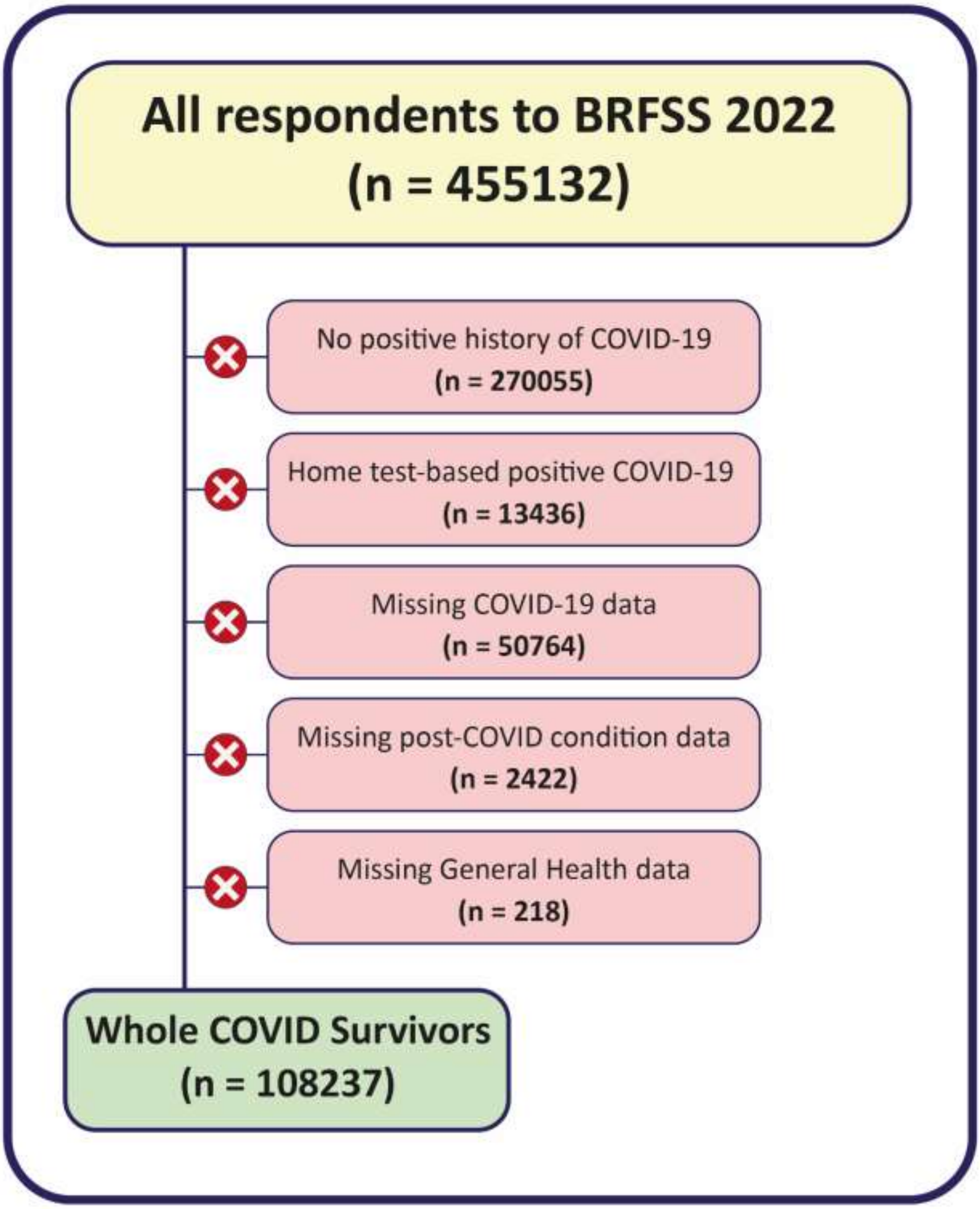

S1 Fig. Flow diagram of the study population. Cross signs show the number of excluded individuals from the study based on each exclusion criterion. BRFSS: Behavioral Risk Factor Surveillance System

S1 Table. The final variables and their corresponding questions based on the BRFSS Questionnaire used in this study

Variable	Variable Name In BRFSS codebook	Variable Questions in the BRFSS Questionnaire	Possible Answers	Variables Categories in BRFSS codebook	Re-Coded Values
Age	**AGE_G**	What is your age?	18-24	18-24	18-34
			25-34	25-34	18-34
			35-44	35-44	35-44
			45-54	45-54	45-64
			55-64	55-64	45-64
			>64	>64	>64
Gender	**SEX**	Are you male or female?	Male	Male	M
			Female	Female	F
BMI	**RFBMI5**	About how much do you weigh without shoes?<br>About how tall are you without shoes?	BMI<=25	BMI<=25	<25
			BMI>25	BMI>25	>25
			Missing	Missing	-
	**BMI5CAT**		<18.5 Kg/m2 (Underweight)	<18.5 Kg/m2 (Underweight)	<18.5
			18.5-24.9 Kg/m2 (Normal weight)	18.5-24.9 Kg/m2 (Normal weight)	18.5-24
			25-29.9 Kg/m2 (Overweight)	25-29.9 Kg/m2 (Overweight)	25-30
			≥30 Kg/m2 (Obese)	≥30 Kg/m2 (Obese)	≥30
			Missing	Missing	-
Marriage status	**MARITAL**	Are you …	Married	Married	Married
			Divorced	Divorced	Single
			Widowed	Widowed	Single
			Separated	separated	Single
			Never married	Never married	Single
			a member of an unmarried couple	member of an unmarried couple	Single
			Missing	Missing	-
Race and ethnicity	**RACEPR1**	Computed based on various self-reported answers to race-related questions.	Non-Hispanic White only	Non-Hispanic White only	White
			Non-Hispanic Black only	Non-Hispanic Black only	Black
			Non-Hispanic Alaskan Native or American Indian only	Non-Hispanic Alaskan Native or American Indian only	American Indian, or Alaskan Native/Others
			non-Hispanic, Asian only	non-Hispanic, Asian only	Asian/Others
			non-Hispanic, Native Hawaiian or other Pacific Islanders only	non-Hispanic, Native Hawaiian or other Pacific Islanders only	Native Hawaiian, or other Pacific Islanders/Others
			non-Hispanic, Multiracial	non-Hispanic, Multiracial	Multiracial/Others
			Hispanic	Hispanic	Hispanic
Education	**EDUCAG**	What is the highest grade or year of school you completed?	Never attended school or only attended kindergarten	Did not finish high school	< High School
			Grades 1 through 8 (Elementary)	Did not finish high school	< High School
			Grades 9 through 11 (Some high school)	Did not finish high school	< High School
			Grade 12 or GED (High school graduate)	Graduated high school	High School
			College 1 year to 3 years (Some college or technical school)	Some college/technical school	> High School
			College 4 years or more (College graduate)	Completed college/technical school	> High School
			Missing	Missing	-
Annual household income	**INCOMG1**	Is your annual household income from all sources …	Less than $10,000?	$<15000	≤$15k or $≤25k
			Less than $15,000? ($10,000 to less than $15,000)	$<15000	≤$15k or $≤25k
			Less than $20,000? ($15,000 to less than $20,000)	$15000–24999	$15–25k or $≤25k
			Less than $25,000? ($20,000 to less than $25,000)	$25000–34999	$25–35k or $≤25-50k
			Less than $35,000? ($25,000 to less than $35,000)	$35000–49999	$35–50k or $≤25-50k
			Less than $50,000? ($35,000 to less than $50,000)	$50000-99999	$≥50-100k
			Less than $75,000? ($50,000 to less than $75,000)	$50000-99999	$≥50-100k
			Less than $100,000? ($75,000 to less than $100,000)	$100000-199999	$≥100-200k or $≥100k
			Less than $150,000? ($100,000 to less than $150,000)	$100000-199999	$≥100-200k or $≥100k
			Less than $200,000? ($150,000 to less than $200,000)	>=$200000	$≥200k or $≥100k
			$200,000 or more	Missing	-
Rural residence	**URBSTAT**		Urban	Urban	Urban

		Respondents were assigned Metropolitan Status Codes based on their landline data; respondents residing in a metropolitan statistical area (urban area and adjacent integrated areas) were categorized as nonrural, and respondents not in a metropolitan statistical area were categorized as rural.	Rural	Rural	Rural
			Missing	blank	-
Stroke belt residence	**Based on _STATE**	In what state do you currently live?	Alabama, Arkansas, Georgia, Louisiana, Mississippi, North Carolina, South Carolina, and Tennessee	Alabama, Arkansas, Georgia, Louisiana, Mississippi, North Carolina, South Carolina, and Tennessee	Resident
			Others	Others	Non-resident
Health insurance	**HLTHPLN**	What is the current primary source of your health insurance ?	Has insurance	Has insurance	Yes
			Don't have insurance	Don't have insurance	No
			Missing	Missing	-
Diabetes	**DIABETE4**	Has a doctor, nurse, or other health professional ever told you that you had any of the following?	Yes	Yes	Yes
			Yes, but female told only during pregnancy	GDM only	No
			No	No history of DM	No
			No, prediabetes or borderline diabetes	Prediabetes	No
			Don't know / Not sure / Refused	Missing	-
Heart disease	**MICHD**	Has a doctor, nurse, or other health professional ever told you that you had angina or coronary heart disease?	MI or CHD+	MI or CHD+	Yes
			MI or CHD-	MI or CHD-	No
			Missing	Missing	-
MI	**CVDINFR4**	Has a doctor, nurse, or other health professional ever told you that you had heart attack also called a myocardial infarction?	Yes	Yes	Yes
			No	No	No
			Missing	Missing	-
Pulmonary disease (**PULMDIS**)	**ASTHMA3**	Has a doctor, nurse, or other health professional ever told you that you had asthma?	Yes	Yes	Yes
			No	No	No
			Missing	Missing	-
	**CHCCOPD3 (COPD, emphysema or chronic bronchitis)**	Has a doctor, nurse, or other health professional ever told you that you had C.O.P.D. (chronic obstructive pulmonary disease), emphysema or chronic bronchitis?	Yes	Yes	Yes
			No	No	No
			Missing	Missing	-
Depression	**ADDEPEV3**	Has a doctor, nurse, or other health professional ever told you that you had a depressive disorder (including depression, major depression, dysthymia, or minor depression)?	Yes	Yes	Yes
			No	No	No
			Missing	Missing	-
Kidney disease	**CHCKDNY2**	Not including kidney stones, bladder infection or incontinence, were you ever told you had kidney disease?	Yes	Yes	Yes
			No	No	No
			Missing	Missing	-
Arthritis	**HAVARTH4**	Has a doctor, nurse, or other health professional ever told you that you had some form of arthritis, rheumatoid arthritis, gout, lupus, or fibromyalgia?	Yes	Yes	Yes
			No	No	No
			Missing	Missing	-
Cancer	**CHCOCNC1**	Has a doctor, nurse, or other health professional ever told you that you had melanoma or any other types of cancer?	Yes	Yes	Yes
			No	No	No
			Missing	Missing	-
Stroke	**CVDSTRK3**	Has a doctor, nurse, or other health professional ever told you that you had stroke?	Yes	Yes	Yes
			No	No	No
			Missing	Missing	-
Smoking status	**SMOKER3**	Have you smoked at least 100 cigarettes in your entire life? Do you now smoke cigarettes every day, some days, or not at all?	Current every day smoker	Current every day smoker	Current Smoker
			Current some day smoker	Current some day smoker	Current Smoker
			Former smoker	Former smoker	Former smoker
			Non-smoker	Non-smoker	Non-smoker
			missing	missing	-
Heavy alcohol drinker	**RFDRHV8**	During the past 30 days, how many days per week or per month did you have at least one drink of any alcoholic beverage such as beer, wine, a malt beverage or liquor? During the past 30 days, on the days when you drank, about how many drinks did you drink on the average?	Yes	Yes	Yes
			No	No	No
			Missing	Missing	-
E-cigarette status	**ECIGNOW2**	Would you say you have never used e-cigarettes or other electronic vaping products in your entire life or now use them every day, use them some days, or used them in the past but do not currently use them at all?	Non-smoker	Current every day smoker	Current Smoker
			Current every day smoker	Current some day smoker	Current Smoker
			Current some day smoker	Former smoker	Former smoker
			Former smoker	Non-smoker	Non-smoker

			Missing	Missing	-
Ever had COVID19	**COVIDPOS**	Has a doctor, nurse, or other health professional ever told you that you tested positive for COVID 19?	Yes	Yes	Yes
			no	No	No
			Missing	Missing	-
PCC+	**COVIDSMP**	Did you have any symptoms lasting 3 months or longer that you did not have prior to having coronavirus or COVID-19?	PCC+	PCC+	Y
			PCC-	PCC-	N
			Missing	Missing	-
Primary PCC	**COVIDPRM**	Which of the following was the primary symptom that you experienced? Was it ….	Tiredness or fatigue	Tiredness or fatigue	Yes/No
			Difficulty thinking or concentrating or forgetfulness/memory problems (sometimes referred to as "brain fog")	Difficulty thinking/concentrating/forgetfulness	Yes/No
			Difficulty breathing/shortness of breath	Difficulty breathing/shortness of breath	Yes/No
			Joint or muscle pain	Joint or muscle pain	Yes/No
			Fast-beating or pounding heart (also known as heart palpitations) or chest pain	Heart palpitation	Yes/No
			Dizziness on standing	Dizziness on standing	Yes/No
			Depression, anxiety, or mood change	Depression, anxiety, or mood change	Yes/No
			Symptoms that get worse after physical or mental activities	Symptoms worsening after physical/mental 8activity	Yes/No
			You did not have any long-term symptoms that limited your activities.	No activity limiting symptom	Yes/No
			Loss of taste or smell	Loss of taste or smell	Yes/No
			Some other symptom	Other symptoms	Yes/No
			Don't know/Not sure 99 Refused	Missing	-
Exercise or physical activity	**TOTINDA**	During the past month, other than your regular job, did you participate in any physical activities or exercises such as running, calisthenics, golf, gardening, or walking for exercise?	Physical activity + 30d	Physical activity + 30d	Physically active
			Physical activity - 30d	Physical activity - 30d	Not physically active
			Missing	Missing	-
Sleep hours a day	**SLEPTIM1**	On average, how many hours of sleep do you get in a 24-hour period?	Numeric	Numeric (1 to 24)	<7 h/day
			Numeric	Numeric (1 to 24)	7-9 h/day
			Numeric	Numeric (1 to 24)	>9 h/day
			Missing	Missing	-
General Health	**GENHLTH**	Would you say that in general your health is:	Excellent	Excellent	Excellent/Good or Better
			Very good	Very good	Very good/ Good or Better
			Good	Good	Good/ Good or Better
			Fair	Fair	Fair/Poor or Fair
			Poor	Poor	Poor/ Poor or Fair
			Missing/Don't know/refused	-	-
Mental Health	**MENT14D**	Thinking about your mental health, which includes stress, depression, and problems with emotions, for how many days during the past 30 days was your mental health not good?	Zero days when mental health not good	Zero days	Zero days
			1-13 days when mental health not good	1-13 days	1-13 days
			14+ days when mental health not good	14+ days	14+ days
			Don't know/Refused/Missing	Missing	-
Physical Health	**PHYS14D**	Thinking about your physical health, which includes physical illness and injury, for how many days during the past 30 days was your physical health not good?	Zero days when physical health not good	Zero days	Zero days
			1-13 days when physical health not good	1-13 days	1-13 days
			14+ days when physical health not good	14+ days	14+ days
			Don't know/Refused/Missing	Missing	-
General health and dialy life	**POORHLTH**	During the past 30 days, for about how many days did poor physical or mental health keep you from doing your usual activities, such as self-care, work, or recreation?	Numeric	Zero days	Zero days
			Numeric	1-13 days	1-13 days
			Numeric	14+ days	14+ days
			Missing	Missing	-

BMI: Body Mass Index
CHD: Coronary Heart Disease
COPD: Chronic Obstructive Pulmonary Disease
DM: Diabetes Mellitus
GDM: Gestational Diabetes Melitus
MI: Myocardial Infarction
PCC: Post-COVID Conditions

S2 Table. The unweighted number of participants with the number of missing data for each variable (supporting data for Table 2).

Feature	Unweighted Frequency Whole COVID survivors (95% CI of percentage)
Respondents, raw unweighted frequency	108237
Respondents, raw U.S. weighted frequency	70002617
**Age**	
Early adulthood (18-34 years old)	24628 (35%)
Early middle age (35-44 years old)	18288 (18.9%)
Late middle age (45-64 years old)	38459 (31%)
Late adulthood (>64 years old)	26862 (15.2%)
Missing	0
**Sex**	
Male	49693 (46.5%)
Female	58544 (53.5%)
Missing	0
**Body Mass Index (BMI)**	
<18.5 Kg/m2 (Underweight)	1347 (1.5%) → 1388 (1.6%)
18.5-24.9 Kg/m2 (Normal weight)	26379 (25.5%) → 27649 (26.8%)
25-29.9 Kg/m2 (Overweight)	34792 (31.2%) → 37794 (34.3%)
≥30 Kg/m2 (Obese)	38928 (34.6%) → 41406 (37.3%)
Missing	6791 (7.1%) → 0
**Marital status** #	
Single	49169 (48.3%)
Married	58310 (50.9%)
Missing	758 (0.7%)
**Race and ethnicity**	
Hispanic	13150 (20.3%)
Non-Hispanic, White	78489 (58.2%)
Non-Hispanic Black	8481 (11.2%)
Non-Hispanic, Asian	2726 (5.3%)
Non-Hispanic, American Indian, or Alaskan Native	2153 (1.2%)
Non-Hispanic, Native Hawaiian, or other Pacific Islanders	584 (0.4%)
non-Hispanic, Multiracial	2654 (3.3%)
Missing	0
**Education**	
less than high school	5645 (9.5%)
High school degree	25800 (27%)
Any college/technical school degree	76434 (63.2%)
Missing	358 (0.3%)
**Annual household income**	
$<15000	4302 (4.2%) → 4391 (4.2%)
$15000–24999	7853 (7.6%) → 8383 (8.2%)
$25000–34999	10388 (10%) → 13587 (13.2%)
$35000–49999	11639 (10.3%) → 14640 (13.4%)
$50000-100000	29381 (25.3%) → 34676 (30.6%)
$100000-200000	21411 (19.4%) → 25002 (22.9%)
$>200000	6648 (6.6%) → 7558 (7.4%)
Missing	16615 (16.6%) → 0
**Rural residence**	
Urban	92140 (92.4%)
Rural	13197 (6%)
Missing	2900 (1.6%)
**Health insurance**	
With health insurance	99566 (89%)
Without health insurance	5206 (6.7%)
Missing	3465 (4.3%)
**Comorbidities**	
Diabetes ɸ	14667 (missing=165, 0.2%)
Heart disease £	8712 (missing=932, 0.9%)
Pulmonary disease ⁋	23576 (missing=521, 0.6%)
Depression	25497 (missing=563, 0.7%)
Kidney disease	4763 (missing=368, 0.3%)
Arthritis	34493 (missing=532, 0.6%)
Cancer	10513 (missing=397, 0.3%)
Stroke	3988 (missing=287, 0.3%)
**Cigarette Smoking Status**	
Current smoker	32613 (27%)
Former smoker	67557 (65.9%)
Non-smoker	7386 (6.6%)
Missing	681 (0.6%)
Heavy alcohol drinker	7299 (missing=1952, 2.3%)
**E-cigarette Smoking Status**	
Current smoker	7542 (9.1%)
Former smoker	21005 (22.3%)
Non-smoker	79320 (68.2%)
Missing	370 (0.4%)
**Daily exercise or physical activity** ¥	
With physical activity	83148 (77.4%)
Without physical activity	24896 (22.4%)
Missing	193 (0.2%)
**Daily Sleep Duration**	
<7 hours/day (short sleepers)	38949 (38.6%)
7-9 hours/day (adequate sleepers)	65298 (57.5%)
>9 hours/day (long sleepers)	3103 (2.9%)
Missing	887 (1%)
**Self-reported General Health**	
Excellent	16857 (16.7%)
Very good	36470 (33%)
Good	35518 (32.4%)
Fair	14840 (13.8%)
Poor	4552 (4%)
Missing	0
**Mental Health**	
No	59183 (51.3%)
1-13 days	30172 (29.1%)
14+ days	17048 (17.6%)
Missing	1834 (2%)
**Physical Health**	
No	60444 (56.5%)
1-13 days	30908 (28.5%)
14+ days	14796 (12.9%)
Missing	2089 (2.1%)
**Daily task interrupted**	
No	33365 (30.6%)
1-13 days	22934 (22.3%)
14+ days	10907 (10.2%)
Missing	41031 (36.9%)

Never-married, divorced, widowed, and unmarried couples are considered single.
ɸ Diabetes does not include prediabetes or gestational Diabetes.
£ Heart disease refers to myocardial infarction, angina, or coronary heart disease.
⁋ Pulmonary diseases refer to asthma, chronic obstructive pulmonary disease, emphysema, and chronic bronchitis.
- Percentages for any given characteristic might not add up to 100% due to the weighted nature of the estimates.
- In BMI and annual income variables, the number before the arrow shows the unweighted frequency before imputation, and the number after the arrow shows the unweighted frequency after imputation.
BMI: Body Mass Index

S3 Table. Self-reported general, mental, and physical health and Daily efficiency among COVID survivors with or without Post-COVID Conditions (Supporting data for Fig 1)

Self-reported General Health	Positive Post-COVID Conditions	Negative Post-COVID Conditions	p-Value
**Excellent**	11.2% (10.4-12)	18.3% (17.8-18.9)	<0.001
**Very good**	27.9% (26.8-29)	34.6% (33.9-35.2)	
**Good**	35.1% (33.9-36.3)	31.6% (31-32.3)	
**Fair**	19.3% (18.3-20.2)	12.2% (11.7-12.7)	
**Poor**	6.5% (5.9-7)	3.2% (3-3.5)	
**Self-reported General Health**	**Positive Post-COVID Conditions**	**Negative Post-COVID Conditions**	**p-Value**
**Favorable**	74.3% (73.2-75.4)	84.5% (84-85.1)	<0.001
**Unfavorable**	25.7% (24.6-26.8)	15.5% (14.9-16)	
**Mental Health**	**Positive Post-COVID Conditions**	**Negative Post-COVID Conditions**	**p-Value**
**No**	42.4% (41.2-43.6)	55.3% (54.6-56)	<0.001
**1-13 days**	32% (30.8-33.2)	29% (28.4-29.6)	
**14+ days**	25.6% (24.5-26.7)	15.7% (15.2-16.2)	
**Physical Health**	**Positive Post-COVID Conditions**	**Negative Post-COVID Conditions**	**p-Value**
**No**	47.7% (46.5-49)	60.6% (59.9-61.3)	<0.001
**1-13 days**	31.8% (30.6-32.9)	28.3% (27.7-29)	
**14+ days**	20.5% (19.5-21.5)	11.1% (10.6-11.5)	
**Daily Efficiency**	**Positive Post-COVID Conditions**	**Negative Post-COVID Conditions**	**p-Value**
**No**	42.2% (40.7-43.6)	50.8% (49.9-51.7)	<0.001
**1-13 days**	36.1% (34.7-37.5)	35.1% (34.2-35.9)	
**14+ days**	21.7% (20.5-22.9)	14.2% (13.5-14.8)	

- Data are presented as frequency and percentages.
- Percentages for any given characteristic may not sum to 100% because table reflect weighted estimates.
- Significant p-values (<0.05) are highlighted.

S4 Table. Self-reported General Health Among COVID Survivors with Different Primary Symptoms (Supporting data for Fig 3)

Self-reported General Health	Fatigue	Brain Fog	Dyspnea	Pain	Heart palpitation	Dizziness	Mood change	Post-exertional Malise	No activity-limiting symptom	Taste/Smell Loss	Others
**Excellent**	9.5% (8.2-10.9)	10.3% (7.7-12.9)	7.4% (5.8-8.9)	6.8% (4.3-9.3)	8.8% (5.7-11.9)	7.5% (2.8-12.1)	2.8% (0.7-4.9)	8.6% (3.1-14.2)	18.6% (14.4-22.8)	17.8% (15.1-20.4)	12.6% (9.9-15.4)
**Very good**	26.1% (23.9-28.2)	30.8% (26.6-35.1)	22.8% (20.5-25.1)	26.2% (21-31.4)	29% (22-36)	24.9% (15.7-34.1)	24.5% (13.9-35.2)	39.2% (28.8-49.7)	35.7% (31.1-40.4)	31.5% (28.8-34.1)	29.6% (25.6-33.6)
**Good**	37.6% (35.2-40)	36.6% (32.7-40.6)	37.9% (35-40.8)	32.9% (27.6-38.2)	34.9% (28.7-41.1)	29.6% (19.6-39.6)	36.4% (25.6-47.1)	30.3% (19.6-41)	31.7% (27.5-36)	32.6% (29.6-35.6)	32% (27.9-36)
**Fair**	19.7% (18-21.4)	18.2% (15.1-21.3)	23.8% (21.4-26.1)	27.1% (21.7-32.5)	20% (14.7-25.3)	31.3% (18.3-44.4)	27.3% (15.3-39.3)	12% (7.1-17)	8.4% (6.2-10.7)	13.6% (11.7-15.6)	19.6% (15-24.2)
**Poor**	7.1% (5.9-8.3)	4% (2.8-5.2)	8.2% (6.8-9.5)	7.1% (4.1-10)	7.3% (3.6-11)	6.7% (3.8-9.7)	9% (4.5-13.5)	9.8% (4.2-15.4)	5.5% (2.1-8.9)	4.5% (3.1-5.9)	6.2% (4.5-7.9)

- Data are presented as frequency and percentages.
- Percentages for any given characteristic may not sum to 100% because table reflect weighted estimates.
- 4.5% of primary PCCs data among COVID survivors was missing.

S5 Table. Prevalence of Unfavorable Self-reported General Health Among COVID Survivors Based on Age and Sex (Supporting data for Fig 4)

Age	Positive Post-COVID Conditions		p-Value	Negative Post-COVID Conditions		p-Value
	Male	Female		Male	Female	
18-34 (Early adulthood)	16.4% (13.8-18.9)	18% (15.8-20.1)	0.162	9.5% (8.4-10.6)	11.6% (10.3-12.8)	<0.001
35-44 (Early middle age)	19.5% (16.1-22.8)	24.7% (21.2-28.2)	0.036	11.9% (10.4-13.4)	14.7% (12.8-16.6)	0.072
45-64 (Late middle age)	30.8% (27.8-33.8)	32.1% (29.7-34.5)	0.641	16.9% (15.7-18.1)	19.3% (17.9-20.7)	0.111
>64 (Late adulthood)	35.5% (31.1-40)	34.6% (30.7-38.5)	0.709	24.3% (22.1-26.5)	23.9% (21.8-26)	0.848

- Data are presented as frequency and percentages.
- Percentages for any given characteristic may not sum to 100% because table reflect weighted estimates.
- Significant p-values (<0.05) are highlighted.

S6 Table. State-wise Prevalence of Unfavorable Self-reported General Health Among COVID Survivors in the US (Supporting data for Fig 5A and 5B)

COVID Survivors with Post-COVID Conditions					
State	Overall Prevalence of Unfavorable General Health	State	Overall Prevalence of Unfavorable General Health	State	Overall Prevalence of Unfavorable General Health
Alabama	21.4% (16.5-26.3)	Louisiana	23.6% (18.6-28.5)	Oklahoma	24.7% (20.4-29)
Alaska	22.4% (16.5-28.2)	Maine	24.5% (18.7-30.3)	Oregon	33.9% (27-40.9)
Arizona	28.3% (22.3-34.4)	Maryland	23.5% (18.4-28.5)	Pennsylvania	20.4% (14.7-26.2)
Arkansas	28.7% (22.5-34.9)	Massachusetts	24.1% (19.4-28.8)	Rhode Island	23.8% (17.3-30.4)
California	29% (22.9-35.1)	Michigan	25.4% (20.7-30.1)	South Carolina	25.3% (20.7-29.9)
Colorado	16% (12.7-19.4)	Minnesota	22.2% (18.3-26)	South Dakota	22.3% (10.5-34)
Connecticut	20.4% (15.6-25.2)	Mississippi	27.9% (22.2-33.7)	Tennessee	20.9% (16-25.9)
Delaware	21.5% (12.8-30.1)	Missouri	24.9% (20.1-29.7)	Texas	29.6% (24.7-34.6)
District of Columbia	20.9% (10.4-31.5)	Montana	28.2% (23.4-33)	Utah	25.7% (21.5-29.9)
Florida	27.3% (21.4-33.1)	Nebraska	24.4% (18.9-29.9)	Vermont	23.7% (15.6-31.8)
Georgia	24% (18.6-29.3)	Nevada	28.9% (19.4-38.3)	Virginia	28% (22.3-33.8)
Hawaii	24.9% (17.3-32.5)	New Hampshire	21% (14.8-27.2)	Washington	28.6% (25.2-32.1)
Idaho	24.8% (20.1-29.5)	New Jersey	22.3% (17.1-27.4)	West Virginia	34.5% (29.2-39.8)
Illinois	19.8% (14.4-25.3)	New Mexico	30.7% (23.3-38.1)	Wisconsin	24.4% (20-28.8)
Indiana	25.5% (21.6-29.4)	New York	25.8% (21.4-30.3)	Wyoming	19.2% (14.1-24.3)
Iowa	22.9% (18.4-27.5)	North Carolina	25.3% (18.6-31.9)	Guam	17.6% (3.6-31.5)
Kansas	21.9% (17.9-26)	North Dakota	16.1% (11.7-20.6)	Puerto Rico	30.5% (24-36.9)
Kentucky	31% (23.2-38.8)	Ohio	28.2% (24.5-31.9)	Virgin Islands	9.7% (-1.5-21)

All COVID Survivors					
State	Overall Prevalence of Unfavorable General Health	State	Overall Prevalence of Unfavorable General Health	State	Overall Prevalence of Unfavorable General Health
Alabama	19.3% (16.5-22)	Louisiana	19.2% (16.7-21.6)	Oklahoma	18.1% (16-20.2)
Alaska	13.7% (11.4-16)	Maine	16.6% (14.3-19)	Oregon	19.1% (16.3-21.9)
Arizona	19.8% (17.1-22.4)	Maryland	15.5% (13.6-17.4)	Pennsylvania	16.8% (13.8-19.9)
Arkansas	21% (18.1-23.8)	Massachusetts	14.8% (12.8-16.8)	Rhode Island	15.4% (12.9-17.9)
California	19.6% (17.3-21.9)	Michigan	16.8% (14.8-18.8)	South Carolina	15.8% (13.9-17.7)
Colorado	12.2% (10.6-13.8)	Minnesota	14.5% (12.9-16)	South Dakota	14.8% (10.8-18.7)
Connecticut	16.1% (13.6-18.7)	Mississippi	23% (20.3-25.7)	Tennessee	17.3% (15-19.6)
Delaware	17.6% (14-21.2)	Missouri	19.3% (17.1-21.6)	Texas	20% (17.7-22.2)
District of Columbia	9.5% (6.6-12.4)	Montana	17.7% (15.5-19.8)	Utah	15.8% (14-17.6)
Florida	17.7% (14.9-20.5)	Nebraska	14.9% (12.7-17)	Vermont	13.9% (11.2-16.7)
Georgia	16.5% (14.3-18.7)	Nevada	23.2% (18.8-27.6)	Virginia	16.6% (14.3-18.9)
Hawaii	12.5% (10.3-14.6)	New Hampshire	15.9% (12.9-19)	Washington	16.8% (15.5-18.2)
Idaho	17.2% (15-19.3)	New Jersey	15.3% (13.3-17.3)	West Virginia	23.9% (21.3-26.6)
Illinois	16.2% (13.5-18.9)	New Mexico	20.8% (17.6-23.9)	Wisconsin	15.1% (13.4-16.8)
Indiana	17.8% (16-19.5)	New York	17.4% (15.8-19.1)	Wyoming	15% (12.3-17.7)
Iowa	15.1% (13.2-16.9)	North Carolina	17.7% (14.8-20.5)	Guam	20.2% (13.1-27.3)
Kansas	13.8% (12.2-15.5)	North Dakota	12.8% (10.7-14.9)	Puerto Rico	22.6% (20.3-25)
Kentucky	18.8% (15.9-21.6)	Ohio	18.8% (17-20.7)	Virgin Islands	13.1% (6.7-19.5)

S7 Table. Sociodemographics, Comorbidities, and Behavioral Determinants of Health among COVID Survivors with Post-COVID Conditions by Their Self-reported General Health

Features	COVID Survivors with Post-COVID Conditions		p-Value
	Good or Better (Favorable) Self-reported General Health (CI95% of percentage)	Fair or Poor (Unfavorable) Self-reported General Health (CI95% of percentage)	
Respondents, raw unweighted frequency	18546	6670	-
Respondents, raw US weighted frequency	11795241	4082399	-
**Age**			
Early adulthood (18-34 years old)	35.6% (34.2-37.1)	21.7% (19.7-23.6)	<0.001
Early middle age (35-44 years old)	21.2% (20-22.4)	18.1% (16-20.2)	
Late middle age (45-64 years old)	31.1% (29.9-32.4)	41.6% (39.2-43.9)	
Late adulthood (>64 years old)	12% (11.2-12.8)	18.6% (16.8-20.4)	
**Sex**			
Male	37.4% (36-38.8)	35% (32.8-37.3)	0.081
Female	62.6% (61.2-64)	65% (62.7-67.2)	
**Body mass index (BMI)**			
<18.5 Kg/m2 (Underweight)	1.7% (1.2-2.2)	1.5% (0.7-2.2)	<0.001
18.5-24.9 Kg/m2 (Normal weight)	24.5% (23.3-25.7)	17.1% (15.4-18.8)	
25-29.9 Kg/m2 (Overweight)	33.2% (31.8-34.5)	26.7% (24.5-29)	
≥30 Kg/m2 (Obese)	40.7% (39.2-42.1)	54.7% (52.2-57.1)	
**Marital status** #			
Single	48.4% (47-49.9)	54.1% (51.6-56.5)	<0.001
Married	51.6% (50.1-53)	45.9% (43.5-48.4)	
**Race and ethnicity**			
Hispanic	18.5% (17.1-19.8)	26.4% (23.8-29)	<0.001
Non-Hispanic, White only	62.7% (61.2-64.2)	53.2% (50.8-55.7)	
Non-Hispanic Black only	9.8% (8.9-10.6)	11.5% (10-13.1)	
Others	9.1% (8.1-10.1)	8.8% (7.5-10.2)	
**Education**			
less than high school	7.3% (6.3-8.3)	18.8% (16.3-21.3)	<0.001
High school degree	25.7% (24.4-26.9)	28.4% (26.4-30.5)	
Higher than high school	67% (65.6-68.5)	52.8% (50.3-55.2)	
**Annual household income (AHHI)**			
$<25k	10.4% (9.6-11.3)	29.1% (26.7-31.5)	<0.001
$25k-50k	26.1% (24.8-27.3)	33.1% (30.8-35.3)	
$50k-100k	34% (32.6-35.3)	24.4% (22.5-26.4)	
$>100k	29.5% (28.2-30.8)	13.4% (11.9-14.9)	
**Rural residence**			
Urban	93.5% (93-94)	92.5% (91.7-93.4)	0.042
Rural	6.5% (6-7)	7.5% (6.6-8.3)	
**Health Insurance Coverage**			
With health insurance	92.8% (92-93.7)	89.4% (87.8-91.1)	<0.001
Without health insurance	7.2% (6.3-8)	10.6% (8.9-12.2)	
**Comorbidities**			
Diabetes ϕ	8.4% (7.7-9.2)	27.5% (25.4-29.6)	<0.001
Heart disease £	4.2% (3.7-4.7)	16.9% (15.4-18.4)	<0.001
Pulmonary disease ⁋	24% (22.8-25.2)	46.2% (43.8-48.7)	<0.001
Depression	28.8% (27.5-30.1)	49.1% (46.6-51.5)	<0.001
Kidney disease	2.7% (2.3-3.1)	10.6% (9.3-11.8)	<0.001
Arthritis	26.8% (25.6-28)	52.6% (50.1-55)	<0.001
Cancer	6.3% (5.6-7.1)	12.2% (10.9-13.4)	<0.001
Stroke	2.4% (2-2.7)	9.6% (8.3-10.8)	<0.001
**Cigarette Smoking Status**			
Non-smoker	64.2% (62.8-65.5)	53% (50.6-55.4)	<0.001
Former smoker	25% (23.8-26.2)	30.8% (28.7-32.9)	
Current smoker	10.8% (9.9-11.7)	16.2% (14.5-17.9)	
Heavy alcohol drinker	7.4% (6.7-8.1)	5.9% (4.8-7)	0.029
**E-cigarette Smoking Status**			
Non-smoker	67.4% (66.1-68.8)	65.4% (63.1-67.7)	0.319
Former smoker	22.8% (21.5-24)	24.1% (22.1-26.2)	
Current smoker	9.8% (8.9-10.6)	10.5% (9-11.9)	
**Daily exercise or physical activity** ¥			
With physical activity	80.7% (79.6-81.8)	56.5% (54-58.9)	<0.001
Without physical activity	19.3% (18.2-20.4)	43.5% (41.1-46)	
**Daily Sleep Duration**			
7-9 hours/day (adequate sleepers)	55.5% (54-56.9)	37.5% (35.1-39.8)	<0.001
<7 hours/day (short sleepers)	42% (40.6-43.5)	56.7% (54.3-59.1)	
>9 hours/day (long sleepers)	2.5% (2-3)	5.9% (4.8-6.9)	

Never-married, divorced, widowed, and unmarried couples are considered as single.
ϕ Diabetes does not include pre-diabetes or gestational Diabetes.
£ Heart disease refers to myocardial infarction, angina, or coronary heart disease.
⁋ Pulmonary diseases refer to asthma, chronic obstructive pulmonary disease, emphysema, and chronic bronchitis.
- Percentages for any given characteristic might not add up to 100% due to the weighted nature of the estimates.
- Significant p-values (<0.05) are highlighted.

S8 Table. Factors Associated with Unfavorable Self-reported General Health Among COVID Survivors with Post-COVID Survivors (Supporting data for Fig 6)

Features	COVID Survivors with Post-COVID Conditions			
	Crude Odds ratio [95%CI]	p-Value	Adjusted Odds ratio [95%CI] [#]	p-Value
**Age**				
Early adulthood (18-34 years old)	Reference	-	Reference	-
Early middle age (35-44 years old)	1.41 (1.17-1.7)	<0.001	1.08 (0.86-1.36)	0.499
Late middle age (45-64 years old)	2.2 (1.9-2.54)	<0.001	1.47 (1.2-1.8)	<0.001
Late adulthood (>64 years old)	2.55 (2.15-3.04)	<0.001	1.17 (0.9-1.52)	0.240
**Sex**				
Male	Reference	-	Reference	-
Female	1.11 (0.99-1.24)	0.081	0.89 (0.77-1.03)	0.116
**Body mass index**				
18.5-24.9 Kg/m2 (Normal weight)	Reference	-	Reference	-
<18.5 Kg/m2 (Underweight)	1.26 (0.7-2.3)	0.442	1.42 (0.77-2.62)	0.261
25-29.9 Kg/m2 (Overweight)	1.15 (0.98-1.36)	0.092	0.97 (0.8-1.17)	0.731
30 Kg/m2 (Obese)	1.92 (1.66-2.22)	<0.001	1.27 (1.06-1.53)	0.008
**Marital status** [#]				
Single	Reference	-	Reference	-
Married	0.8 (0.71-0.9)	<0.001	1.03 (0.88-1.21)	0.680
**Race and ethnicity**				
Hispanic	Reference	-	Reference	-
Non-Hispanic, White only	0.59 (0.5-0.7)	<0.001	0.52 (0.42-0.63)	<0.001
Non-Hispanic Black only	0.83 (0.65-1.05)	0.115	0.74 (0.55-0.99)	0.044
Others	0.68 (0.53-0.88)	0.003	0.58 (0.4-0.82)	0.002
**Education**				
less than high school	Reference	-	Reference	-
High school degree	0.43 (0.34-0.55)	<0.001	0.74 (0.56-0.99)	0.039
Higher than high school	0.31 (0.24-0.38)	<0.001	0.65 (0.49-0.86)	0.003
**Annual household income (AHHI)**				
$<25k	Reference	-	Reference	-
$25k-50k	0.45 (0.38-0.54)	<0.001	0.64 (0.52-0.8)	<0.001
$50k-100k	0.26 (0.22-0.31)	<0.001	0.44 (0.35-0.55)	<0.001
$>100k	0.16 (0.13-0.2)	<0.001	0.32 (0.24-0.41)	<0.001
**Rural residence**				
Urban	Reference	-	Reference	-
Rural	1.17 (1.01-1.36)	0.042	1.04 (0.85-1.26)	0.734
**Health Insurance Coverage**				
With health insurance	Reference	-	Reference	-
Without health insurance	1.53 (1.23-1.9)	<0.001	1.3 (0.97-1.73)	0.078
**Comorbidities**				
Diabetes [ϕ]	4.12 (3.57-4.75)	<0.001	2.29 (1.88-2.79)	<0.001
Heart disease [£]	4.64 (3.96-5.43)	<0.001	2.01 (1.63-2.49)	<0.001
Pulmonary disease [⁋]	2.72 (2.41-3.06)	<0.001	1.98 (1.71-2.31)	<0.001
Depression	2.38 (2.12-2.67)	<0.001	1.83 (1.58-2.13)	<0.001
Kidney disease	4.2 (3.43-5.13)	<0.001	1.59 (1.17-2.17)	0.003
Arthritis	3.03 (2.69-3.41)	<0.001	1.72 (1.48-2)	<0.001
Cancer	2.04 (1.73-2.42)	<0.001	1.33 (1.09-1.62)	0.006
Stroke	4.38 (3.54-5.43)	<0.001	1.6 (1.16-2.22)	0.005
**Cigarette Smoking Status**				
Non-smoker	Reference	-	Reference	-
Former smoker	1.49 (1.31-1.68)	<0.001	1.1 (0.93-1.3)	0.251
Current smoker	1.81 (1.54-2.14)	<0.001	0.99 (0.78-1.24)	0.910
Heavy alcohol drinker	0.78 (0.62-0.97)	0.029	0.93 (0.72-1.21)	0.612
**E-cigarette Smoking Status**				
Non-smoker	Reference	-	Reference	-
Former smoker	1.09 (0.96-1.25)	0.192	1.1 (0.92-1.31)	0.302
Current smoker	1.1 (0.91-1.33)	0.319	1.3 (1.02-1.65)	0.037
**Daily exercise or physical activity** [¥]				
With physical activity	Reference	-	Reference	-
Without physical activity	3.22 (2.85-3.64)	<0.001	1.94 (1.66-2.27)	<0.001
**Daily Sleep Duration**				
7-9 hours/day (adequate sleepers)	Reference	-	Reference	-
<7 hours/day (short sleepers)	2 (1.77-2.25)	<0.001	1.55 (1.34-1.8)	<0.001
>9 hours/day (long sleepers)	3.47 (2.6-4.63)	<0.001	2.15 (1.53-3.03)	<0.001

Adjusted for sociodemographics (Age, Sex, Marital Status, Race and Ethnicity, Education, AHHI, Rural residence, and Health insurance coverage), comorbidities (Diabetes, Cardiovascular diseases, Pulmonary diseases, Depression, Kidney disorders, Cancer, Arthritis, Obesity), and behavioral determinants of health (Cigarette smoking, E-cigarette smoking, Heavy alcohol consumption, Exercising, and Habitual sleep duration).
- Significant p-values (< 0.05) are highlighted.
